# Understanding the Emergence of Multidrug-Resistant *Candida*: Using Whole-Genome Sequencing to Describe the Population Structure of *Candida haemulonii* Species Complex

**DOI:** 10.3389/fgene.2020.00554

**Published:** 2020-06-10

**Authors:** Lalitha Gade, Jose F. Muñoz, Mili Sheth, Darlene Wagner, Elizabeth L. Berkow, Kaitlin Forsberg, Brendan R. Jackson, Ruben Ramos-Castro, Patricia Escandón, Maribel Dolande, Ronen Ben-Ami, Andrés Espinosa-Bode, Diego H. Caceres, Shawn R. Lockhart, Christina A. Cuomo, Anastasia P. Litvintseva

**Affiliations:** ^1^Mycotic Diseases Branch, Centers for Disease Control and Prevention, Atlanta, GA, United States; ^2^Infectious Disease and Microbiome Program, Broad Institute, Cambridge, MA, United States; ^3^Biotechnology Core Facility Branch, DSR/NCEZID - Centers for Disease Control and Prevention, Atlanta, GA, United States; ^4^IHRC, Inc., Atlanta, GA, United States; ^5^Department of Clinical and Molecular Microbiology, Instituto Conmemorativo Gorgas de Estudios de La Salud, Panama City, Panama; ^6^Grupo de Microbiologia, Instituto Nacional de Salud, Bogotá, Colombia; ^7^Departamento de Micología, Instituto Nacional de Higiene Rafael Rangel, Caracas, Venezuela; ^8^Tel Aviv Sourasky Medical Center, Sackler School of Medicine, Tel Aviv University, Tel Aviv, Israel; ^9^DGHP (Division of Global Health Protection), Central America Region Office, Centers for Disease Control and Prevention, Atlanta, GA, United States; ^10^Center of Expertise in Mycology Radboudumc/CWZ, Nijmegen, Netherlands

**Keywords:** Candida, haemulonii, duobushaemulonii, pseudohaemulonii, vulturna

## Abstract

The recent emergence of a multidrug-resistant yeast, *Candida auris*, has drawn attention to the closely related species from the *Candida haemulonii* complex that include *C. haemulonii, Candida duobushaemulonii, Candida pseudohaemulonii*, and the recently identified *Candida vulturna*. Here, we used antifungal susceptibility testing and whole-genome sequencing (WGS) to investigate drug resistance and genetic diversity among isolates of *C. haemulonii* complex from different geographic areas in order to assess population structure and the extent of clonality among strains. Although most isolates of all four species were genetically distinct, we detected evidence of the in-hospital transmission of *C. haemulonii* and *C. duobushaemulonii* in one hospital in Panama, indicating that these species are also capable of causing outbreaks in healthcare settings. We also detected evidence of the rising azole resistance among isolates of *C. haemulonii* and *C. duobushaemulonii* in Colombia, Panama, and Venezuela linked to substitutions in *ERG11* gene as well as amplification of this gene in *C. haemulonii* in isolates in Colombia suggesting the presence of evolutionary pressure for developing azole resistance in this region. Our results demonstrate that these species need to be monitored as possible causes of outbreaks of invasive infection.

## Introduction

Yeasts from *Candida haemulonii* complex that include *C. haemulonii, Candida duobushaemulonii, Candida pseudohaemulonii*, and the recently identified *Candida vulturna* (Sipiczki and Tap, 2016) are often misidentified as *Candida auris*, especially in laboratories that do not have access to DNA sequencing and matrix-assisted laser desorption/ionization time of flight mass spectroscopy (MALDI-TOF MS) (Hou et al., 2016; Araúz et al., 2018). Together with *C. lusitaniae*, another infrequent cause of candidemia, these four species belong to the Metschnikowiaceae family, which includes species that are often resistant to antifungal drugs (Jackson et al., 2019). Recent comparative genomic analysis identified the substantial amount of conservation and synteny among genomes of *C. auris, C. haemulonii, C. duobushaemulonii*, and *C. pseudohaemulonii* as well as similar expansions of the oligopeptide transporters and lipase gene families (Muñoz et al., 2018). These shared genomic features suggest similarities in ecology and physiology of these species and raise a possibility that they may also start emerging as drug-resistant pathogens in human populations.

In contrast to *C. auris*, which was described in 2009 from an external ear canal from a Japanese patient (Satoh et al., 2009; Chowdhary et al., 2013), *C. haemulonii* was first isolated from a fish off the coast of the Bahamas in 1962 and later isolated from a dolphin and seawater off the coast of Portugal (Van Uden and Kolipinski, 1962). The first clinical isolate was described in 1984 from blood of a patient with renal failure, and since then, isolates of *C. haemulonii* have been infrequently but regularly reported in patients causing wound and other types of infections (Gargeya et al., 1991; Cendejas-Bueno et al., 2012; Hou et al., 2016). *C. pseudohaemulonii* and *C. duobushaemulonii* were identified in 2006 and 2012, respectively, as the distinct lineages within *C. haemulonii* species complex based on the phylogenetic analysis of rDNA intragenic spacer region (ITS) (Cendejas-Bueno et al., 2012). Finally, in 2016, *C. vulturna* was identified as a distinct species most closely related to *C. duobushaemulonii* based on the phylogenetic analysis of rDNA locus (Sipiczki and Tap, 2016). The first isolate of this species was isolated from flowers in the Philippines, and later it was isolated as a cause of human candidemia (Sipiczki and Tap, 2016).

Subsequent studies identified phenotypical and clinical features associated with different species. Specifically, most strains of *C. haemulonii* cannot grow at 37°C, whereas most *C. duobushaemulonii* and *C. pseudohaemulonii* grow well at body temperature, and most strains of *C. auris* can grow up to 42°C (Ben-Ami et al., 2017). Compared with *C. auris* and *C. albicans, C. haemulonii* is less virulent in a murine model of infection (Ben-Ami et al., 2017). In humans, yeasts from *C. haemulonii* complex are primarily known to cause wound infections or colonization although a few cases of invasive blood infections have also been described (Ramos et al., 2015; Hou et al., 2016; Ben-Ami et al., 2017). Conversely, *C. auris* frequently causes invasive infections although the first isolates of *C. auris* were isolated from ear infections, and the East Asian clade of *C. auris* (Clade II) are commonly isolated from the external ear canal (Jackson et al., 2019). *C. auris* and the four species from the *C. haemulonii* complex are known for their resistance to antifungal drugs, especially to amphotericin B and azoles; however, resistance to echinocandins is rare (Ramos et al., 2015; Hou et al., 2016).

Unlike *C. auris*, which is known to colonize human skin and cause rampant healthcare-associated outbreaks in numerous countries, other species from *C. haemulonii* species complex have been associated with relatively few suspected outbreaks and clusters. In 2007, an outbreak of fungemia caused by *C. haemulonii* resistant to amphotericin B, fluconazole, and itraconazole was described among four patients in a neonatal unit in Kuwait (Khan et al., 2007). Although this outbreak occurred before *C. auris* and *C. duobushaemulonii* species were described, ITS sequences from the isolates deposited into NCBI identify the isolates from this outbreak as *C. haemulonii sensu stricto* (Isla et al., 2017). In 2016, a cluster of three *C. haemulonii* wound infections was identified in an Israeli hospital overlapping in time with a *C. auris* outbreak (Ben-Ami et al., 2017). In 2017, an unusual number of *C. duobushaemulonii* and *C. auris* infections were identified in Panama City, Panama, and a subsequent epidemiological investigation confirmed outbreaks of both species (Araúz et al., 2018). The goal of this study was to investigate the genetic relationships and drug-resistance profiles among isolates of the *C. haemulonii* complex from different countries and healthcare facilities to determine genetic diversities in different populations and to examine possible evidence of transmission if it existed.

## Methods

### Isolates

Isolates were received in the Mycotic Diseases Branch Reference Laboratory at the CDC for routine fungal identification or as part of ongoing fungal disease surveillance. Upon arrival, isolates were identified by sequencing of the ITS2 region of the rDNA and MALDI-TOF MS (Bruker, Bremen, Germany) using a CDC-developed database MicrobeNet (https://www.cdc.gov/microbenet/index.html). Isolates were stored in 20% glycerol at −70°C. When available, limited metadata, such as geographic region or site of the infection, were collected.

### Whole-Genome Sequencing (WGS)

DNA was extracted using the ZR Fungal/Bacterial DNA MiniPrep kit (Zymo Research, Irvine, CA, USA) according to the manufacturer's instructions. Genomic libraries were constructed and barcoded using the NEBNext Ultra DNA Library Prep kit for Illumina (New England Biolabs, Ipswich, MA, USA) following the manufacturer's instructions. Libraries were sequenced on either the Illumina HiSeq 2500 platform (Illumina, San Diego, CA, USA) using the HiSeq Rapid SBS Kit v2 500 cycles or the MiSeq platform using the MiSeq Reagent Kit v2 500 cycles or Illumina MiSeq Reagent Kit v3 (600 cycles). HiSeq and MiSeq v2 500-cycle kits generated 251 bp paired reads, whereas MiSeq v3 600-cycle kits generated 301 bp paired reads.

### Single Nucleotide Polymorphism (SNP) Analysis

Paired-end sequences that had at least 50X coverage were used for downstream analyses. Read quality was assessed using FastQC v0.11.5 (http://www.bioinformatics.babraham.ac.uk/projects/fastqc/), and for filtering low-quality sequences, PRINSEQ v0.20.3 (http://prinseq.sourceforge.net/manual.html) was performed using the following command: “-trim_left 15 -trim_qual_left 20 -trim_qual_right 20 -min_len 100 -min_qual_mean 25 -derep 14.” For identifying SNPs, paired-end reads of each species were aligned using BWA mem v0.7.12 ((Li and Durbin, 2009)) to their respective previously published assemblies [*C. haemulonii* strain B11899, GenBank accession PKFO00000000 (Chow et al., 2018a); *C. duobushaemulonii* strain B09383, GenBank accession PKFP00000000 (Chow et al., 2018b); *C. pseudohaemulonii* strain B12108, GenBank accession PYFQ00000000 (Muñoz et al., 2018); and *C. vulturna* strain CBS14366 BioProject PRJNA560499 (Navarro-Muñoz et al., 2019)]. SNPs were identified and filtered using the publicly available pipeline NASP (http://tgennorth.github.io/NASP/) to remove positions that had <10x coverage, <90% variant allele calls, or that were identified by Nucmer (Kurtz et al., 2004) as being within duplicated regions in the reference (Supplementary Table 1).

In addition, we performed variant identification to each of the four mapped species using GATK v3.7 (https://gatk.broadinstitute.org/hc/en-us) with the haploid mode and GATK tools. Sites were filtered with variant filtration using “QD < 2.0 || FS > 60.0 || MQ < 40.0.” Genotypes were filtered if the minimum genotype quality <50, percentage alternate allele <0.8, or depth <10 (https://github.com/broadinstitute/broad-fungalgroup/blob/master/scripts/SNPs/filterGatkGenotypes.py). For the *C. haemulonii* species complex phylogeny, SNPs from the representative isolates were identified by aligning reads to B8441 *C. auris* reference genome assembly, GenBank accession PEKT00000000.2 (Muñoz et al., 2018). To investigate genetic relationships within each species, phylogenetic trees were constructed by identifying SNPs by aligning reads to the reference genome assemblies of each corresponding species.

### Phylogenetic and Population Genetic Analyses

For phylogenetic analysis, maximum parsimony phylogenies were constructed using the subtree-pruning-regrafting (SPR) algorithm and bootstrapped using 500 reiterations in MEGA7.0 (Kumar et al., 2018) and visualized in Interactive Tree of Life (iTOL) v4 (Letunic and Bork, 2019). All positions containing gaps and missing data were eliminated. The average genome-wide nucleotide diversity (π) and Tajima's D were calculated from the GATK SNPs set using PopGenome v2.6.1 R package (https://www.rdocumentation.org/packages/PopGenome/versions/2.7.2) using 5 kb sliding windows. Mating type locus (MTLa and MTLα) was determined using the normalized average read depth at the locus from aligned BAM files for each isolate mapped to the corresponding reference genomes. Genomic regions that exhibit copy number variation (CNV) were identified using normalized read depth.

### Antifungal Susceptibility Testing

Antifungal susceptibility testing was performed as outlined by Clinical and Laboratory Standards Institute (CLSI) standard M27 (CLSI, 2008). However, for several slow-growing isolates of *C. haemulonii*, the plates were read at 48 h as suggested in CLSI standard M27. Etests (BioMérieux, Marcy l'Etoile, France) were used for amphotericin B. Custom-prepared frozen panels (Trek Diagnostics, Thermo Fisher Scientific, Oakwood Village, OH) were used for the echinocandins (anidulafungin, caspofungin, and micafungin) and the azoles (fluconazole, voriconazole, itraconazole, posaconazole, and isavuconazole).

### Identification of Mutations Associated With Elevated MICs

The annotated GATK VCF files were used to determine the genotype of known mutation sites in *ERG11, FKS1*, and other genes of interest (Supplementary Table 2) (Arendrup and Patterson, 2017; Berkow and Lockhart, 2017) using SnpEff v4.3T (http://snpeff.sourceforge.net/).

## Results

### Isolates Description

Between 2011 and 2018, 38 isolates of *C. haemulonii* from Colombia (*N* = 11), Israel (*N* = 3), Panama (*N* = 4), Venezuela (*N* = 7), and the United States (*N* = 13); 55 isolates of *C. duobushaemulonii* from Colombia (*N* = 6), Guatemala (*N* = 2), Panama (*N* = 14), Venezuela (*N* = 1), and the United States (*N* = 32); and six isolates of *C. pseudohaemulonii* from Colombia (*N* = 2), Panama (*N* = 2), Venezuela (*N* = 1), and the United States (*N* = 1) were received (Table 1). In addition, five isolates from Colombia (*N* = 2), Panama (*N* = 2), and the United States (*N* = 1) that were identified by MALDI-TOF as closely resembling *C. duobushaemulonii*, which the subsequent analysis confirmed as *C. vulturna*, were also included (Table 1). We included two historic isolates from the Mycotic Diseases Branch culture collection: B10441 (CBS5149) strain of *C. haemulonii* isolated from a fish in 1962 and B10440 strain of *C. duobushaemulonii* isolated from a foot ulcer of a patient in 1990.

**Table 1:** List of isolates included in the study.

**Isolate**	**Species**	**Origin**	**Year collected**	**Location**	**Mating type**
B10441	*C. haemulonii*	Fish (CBS 5149)	1962	Florida, USA	α
B11786	*C. haemulonii*	Blood	2016	Colombia	α
B11792	*C. haemulonii*	Blood	2016	Colombia	α
B11798	*C. haemulonii*	Blood	2016	Colombia	α
B11803	*C. haemulonii*	Blood	2016	Colombia	α
B11898	*C. haemulonii*	Wound	2014	Israel	α
B11899	*C. haemulonii*	Wound	2015	Israel	α
B11900	*C. haemulonii*	Wound	2015	Israel	α
B12068	*C. haemulonii*	Bronchial Wash	2018	Alabama, USA	α
B12109	*C. haemulonii*	Blood	2011	Venezuela	α
B12112	*C. haemulonii*	Blood	2012	Venezuela	α
B12126	*C. haemulonii*	Blood	2013	Venezuela	α
B12127	*C. haemulonii*	Blood	2014	Venezuela	α
B12128	*C. haemulonii*	Blood	2014	Venezuela	α
B12129	*C. haemulonii*	Blood	2014	Venezuela	α
B12185	*C. haemulonii*	Blood	2016	Venezuela	α
B12201	*C. haemulonii*	Foot	2016	Michigan, USA	α
B12343	*C. haemulonii*	Wound	2016	Georgia, USA	α
B12615	*C. haemulonii*	Wound (big toe)	2017	Indiana, USA	α
B12643	*C. haemulonii*	Wound	2017	Washington, USA	α
B12989	*C. haemulonii*	Foot	2017	Wisconsin, USA	α
B13065	*C. haemulonii*	Vaginal secretion	2016	Panama	α
B13067	*C. haemulonii*	Toenail	2017	Panama	α
B13068	*C. haemulonii*	Blood	2017	Panama	α
B13081	*C. haemulonii*	Catheter	2017	Panama	α
B13273	*C. haemulonii*	Bone	2017	Wisconsin, USA	α
B13444	*C. haemulonii*	Wound	2017	Tennessee, USA	α
B13704	*C. haemulonii*	Bone	2017	Florida, USA	α
B13909	*C. haemulonii*	Blood	2017	Virginia, USA	α
B15318	*C. haemulonii*	Unknown	2018	Hawaii, USA	α
B15393	*C. haemulonii*	Blood	2016	Colombia	α
B15394	*C. haemulonii*	Blood	2016	Colombia	α
B15400	*C. haemulonii*	Urine	2017	Colombia	α
B15401	*C. haemulonii*	Nail	2017	Colombia	α
B15406	*C. haemulonii*	Blood	2017	Colombia	α
B15408	*C. haemulonii*	Peritoneal fluid	2017	Colombia	α
B15409	*C. haemulonii*	Blood	2018	Colombia	α
B16299	*C. haemulonii*	Foot (tissue)	2018	Florida, USA	α
B09383	*C. duobushaemulonii*	Blood	2011	Tennessee, USA	α
B10440	*C. duobushaemulonii*	Foot ulcer	1990	Alabama, USA	α
B11839	*C. duobushaemulonii*	Blood	2016	Mississippi, USA	α
B12075	*C. duobushaemulonii*	Scalp Tissue	2016	Kentucky, USA	α
B12111	*C. duobushaemulonii*	Blood	2011	Venezuela	α
B12240	*C. duobushaemulonii*	Foot	2016	Florida, USA	α
B12437	*C. duobushaemulonii*	Unknown	Missing	Guatemala	α
B12484	*C. duobushaemulonii*	Skin	2017	Florida, USA	α
B12492	*C. duobushaemulonii*	Ear fluid	2017	Florida, USA	α
B12539	*C. duobushaemulonii*	Skin	2017	Florida, USA	α
B12593	*C. duobushaemulonii*	Wound (foot)	2017	Florida, USA	α
B12594	*C. duobushaemulonii*	Wound (toe)	2017	Mississippi, USA	α
B12613	*C. duobushaemulonii*	Wound (toe)	2017	Florida, USA	α
B12614	*C. duobushaemulonii*	Wound (big toe)	2017	Indiana, USA	α
B12709	*C. duobushaemulonii*	Blood	2017	Alabama, USA	α
B12845	*C. duobushaemulonii*	Wound (leg)	2017	Washington, USA	α
B12848	*C. duobushaemulonii*	Wound (toe)	2017	Washington, USA	α
B12972	*C. duobushaemulonii*	Wound (foot)	2017	Florida, USA	α
B12985	*C. duobushaemulonii*	Wound (leg)	2017	Washington, USA	α
B12987	*C. duobushaemulonii*	Wound (toe)	2017	Nebraska, USA	α
B12988	*C. duobushaemulonii*	Nail	2017	Wisconsin, USA	α
B13055	*C. duobushaemulonii*	Blood-tip CVC	2016	Panama	α
B13056	*C. duobushaemulonii*	Blood-tip CVC	2016	Panama	α
B13057	*C. duobushaemulonii*	Blood-tip CVC	2016	Panama	α
B13058	*C. duobushaemulonii*	Toenail scraping	2016	Panama	α
B13059	*C. duobushaemulonii*	Blood	2016	Panama	α
B13063	*C. duobushaemulonii*	Blood	2016	Panama	α
B13066	*C. duobushaemulonii*	Blood	2016	Panama	α
B13071	*C. duobushaemulonii*	Urine	2017	Panama	α
B13072	*C. duobushaemulonii*	Toenail	2017	Panama	α
B13073	*C. duobushaemulonii*	Blood	2017	Panama	α
B13076	*C. duobushaemulonii*	Skin	2017	Panama	α
B13088	*C. duobushaemulonii*	Blood	2017	Panama	α
B13089	*C. duobushaemulonii*	Open fracture	2017	Panama	α
B13091	*C. duobushaemulonii*	Blood	2017	Panama	α
B13267	*C. duobushaemulonii*	Wound (toe)	2017	Oklahoma, USA	α
B13331	*C. duobushaemulonii*	Tissue (foot)	2017	Florida, USA	α
B13465	*C. duobushaemulonii*	Wound	2017	Washington, USA	α
B13467	*C. duobushaemulonii*	Unknown	Missing	Guatemala	α
B13908	*C. duobushaemulonii*	Wound	2017	Virginia, USA	α
B14153	*C. duobushaemulonii*	Bone	2018	Pennsylvania, USA	α
B14185	*C. duobushaemulonii*	Urine	2018	Florida, USA	α
B14283	*C. duobushaemulonii*	Tissue	2018	Virginia, USA	α
B15056	*C. duobushaemulonii*	Wound	2018	Indiana, USA	α
B15179	*C. duobushaemulonii*	Bone	2018	Ohio, USA	α
B15319	*C. duobushaemulonii*	Bone	2018	Tennessee, USA	α
B15360	*C. duobushaemulonii*	Blood	2018	Texas, USA	α
B15396	*C. duobushaemulonii*	Eye secretion	2017	Colombia	α
B15397	*C. duobushaemulonii*	Blood	2017	Colombia	α
B15399	*C. duobushaemulonii*	Blood	2017	Colombia	α
B15403	*C. duobushaemulonii*	Blood	2017	Colombia	α
B15407	*C. duobushaemulonii*	Blood	2017	Colombia	α
B15410	*C. duobushaemulonii*	Tissue	2018	Colombia	α
B16327	*C. duobushaemulonii*	Unknown	2018	New York, USA	α
B16366	*C. duobushaemulonii*	Tissue	2018	Virginia, USA	α
B12108	*C. pseudohaemulonii*	Blood	2011	Venezuela	a
B12384	*C. pseudohaemulonii*	Blood	2016	Georgia, USA	a
B13062	*C. pseudohaemulonii*	Blood	2016	Panama	a
B13064	*C. pseudohaemulonii*	Blood	2017	Panama	a
B15395	*C. pseudohaemulonii*	Blood	2017	Colombia	a
B15405	*C. pseudohaemulonii*	Blood	2017	Colombia	a
B13074	*C. vulturna*	Endothelial secretion	2017	Panama	a
B13075	*C. vulturna*	Mesh Wound	2017	Panama	a
B14309	*C. vulturna*	Wound	2018	Indiana, USA	α
B15411	*C. vulturna*	Gastric acid	2018	Colombia	a
B15412	*C. vulturna*	Blood	2018	Colombia	a

In the United States, 91% of *C. haemulonii* and 87% of *C. duobushaemulonii* were isolated from wounds, bones, and other non-invasive sites, and the remaining isolates were from blood (Table 2). A different distribution of cases was observed in Latin America: 77% of *C. haemulonii* and 67% of *C. duobushaemulonii* from Latin American countries (Colombia, Panama, and Venezuela combined) were from blood, and the remaining isolates were from wounds and non-invasive sites. All isolates of *C. haemulonii* from Israel were from wounds. All six *C. pseudohaemulonii* isolates were from blood (Table 2). One isolate of *C. vulturna* was from blood, and the other four were wounds and other non-invasive sites (Table 2).

**Table 2:** Country and specimen sources of *Candida haemulonii* species complex isolates.

**Organism (*n*)**	**Country (*n*)**	**Blood**	**Wound and other non-invasive sites**
*C. haemulonii* (38^∧^)	USA (11)	1 (9%)	10 (91%)
	Latin America (22)	17 (77%)	5 (23%)
*C. duobushaemulonii* (55^∧^)	USA (31)	4 (13%)	27 (87%)
	Latin America (21)	14 (67%)	7 (33%)
*C. pseudohaemulonii* (6)	USA (1)	1 (100%)	0 (0%)
	Latin America (5)	5 (100%)	0 (0%)
*C. vulturna* (5)	USA (1)	0 (0%)	1 (100%)
	Latin America (4)	1 (25%)	3 (75%)

∧ *Five C. haemulonii and 3 C. duobushaemulonii isolates were excluded, because for 4 isolates, the infection site information was unavailable, one isolate was from fish and only 3 isolates were available from Israel*.

### Phylogenetic Relationships Within the Species Complex

**Figure 1:**
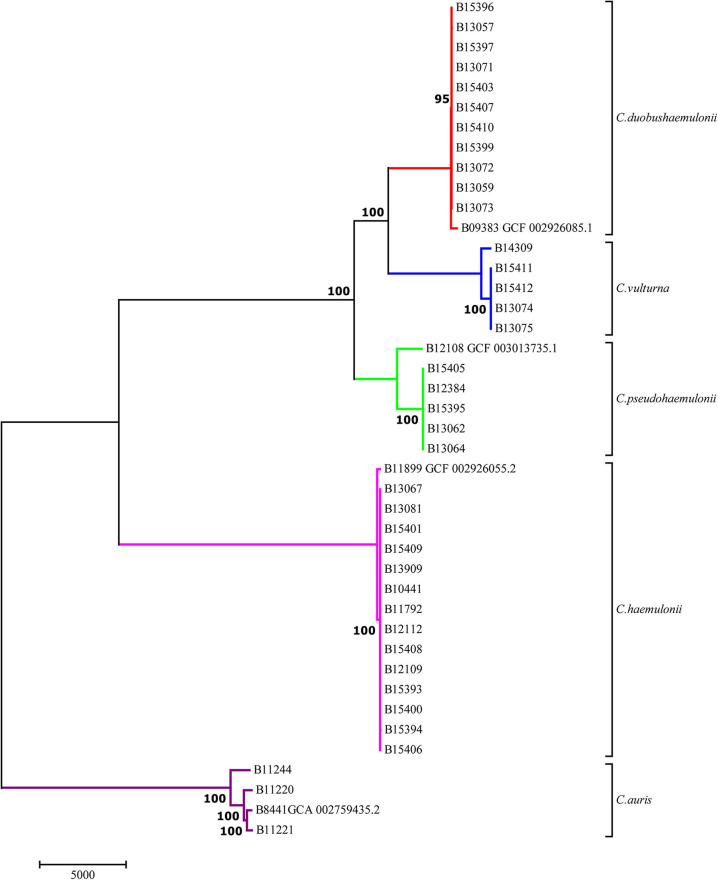
Maximum parsimony tree illustrating the phylogenetic relationships among species from *C. haemulonii* complex constructed using 56,023 SNPs called against B8441 *C. auris* reference genome (GenBank accession PEKT00000000.2). Numbers show bootstrap support for the branches. Scale bar shows pairwise SNPs.

**Figure 2:**
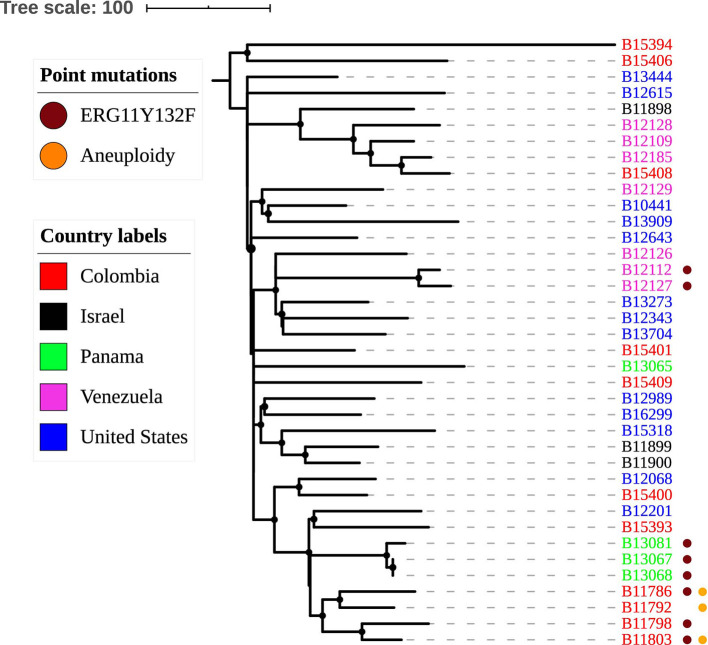
Genetic relationships among *C. haemulonii*. The maximum parsimony tree was constructed using 3586 SNPs called against B11899 *C. haemulonii* reference genome (GenBank accession PKFO00000000). Scale bar shows pairwise SNPs.

Phylogenetic analysis using SNPs called against the *C. auris* reference strain B8441 (Muñoz et al., 2018) confirmed previous observations that *C. duobushaemulonii* is closely related to *C. pseudohaemulonii*. This analysis also identified a subgroup of isolates from Colombia, Panama, and the Unites States, which was identified by MALDI-TOF as closely resembling *C. duobushaemulonii*, which formed a distinct monophyletic branch on the phylogenetic tree (Figure 1). Comparison with a recently assembled genome identified these strains as *C. vulturna* (Navarro-Muñoz et al., 2019) (**Figure 4B**). Conversely, isolates identified by MALDI-TOF as *C. haemulonii* var. *vulnera*, a previously recognized variety within the *C. haemulonii* complex (Cendejas-Bueno et al., 2012), were intermixed with other *C. haemulonii* strains and did not form a phylogenetically distinct cluster (Figures 1, 2).

### Genetic Diversity Among *C. haemulonii*

Genetic relationships among *C. haemulonii* isolates are shown in Figure 2 and at https://microreact.org/project/Candida_haemulonii. The average pairwise difference between the isolates was 214 SNPs (range 0–399), and there was no distinct phylogeographic population structure. Most isolates were genetically distinct; however, three isolates, B13067, B13068, and B13081, were different from each other by fewer than 27 SNPs and formed a small, well-supported cluster in the phylogenetic tree based on bootstrap analysis (Figure 2). These three isolates were recovered from different patients treated at the same hospital in Panama City, Panama, which reported contemporaneous outbreaks of *C. duobushaemulonii* and *C. auris* (Araúz et al., 2018). B13067 was isolated from a toenail of a patient in November 2016, and B13068 was from the blood of another patient isolated in December 2016; the two isolates were genetically identical (0 SNPs). The other isolate from this cluster, B13081, was recovered from a central venous catheter in March 2017. In addition, two isolates from Venezuela, B12112 and B12127, differed by only 32 SNPs although they were isolated from patients treated in different healthcare facilities in the city of Valencia (Figure 2). The rest of the isolates from these and other countries were different from each other by >69 SNPs. All isolates from the United States were genetically distinct. Interestingly, B10441, recovered in 1962 from a seawater fish, was only 176 SNP different from B13909, isolated in 2017 from a patient's blood. All *C. haemulonii* isolates were mating type alpha. The average genome-wide nucleotide diversity (π) was 2.59e-05, and the average Tajima's D estimate was −0.97, which is consistent with recent population expansion.

### Genetic Diversity Among *C. duobushaemulonii*

**Figure 3:**
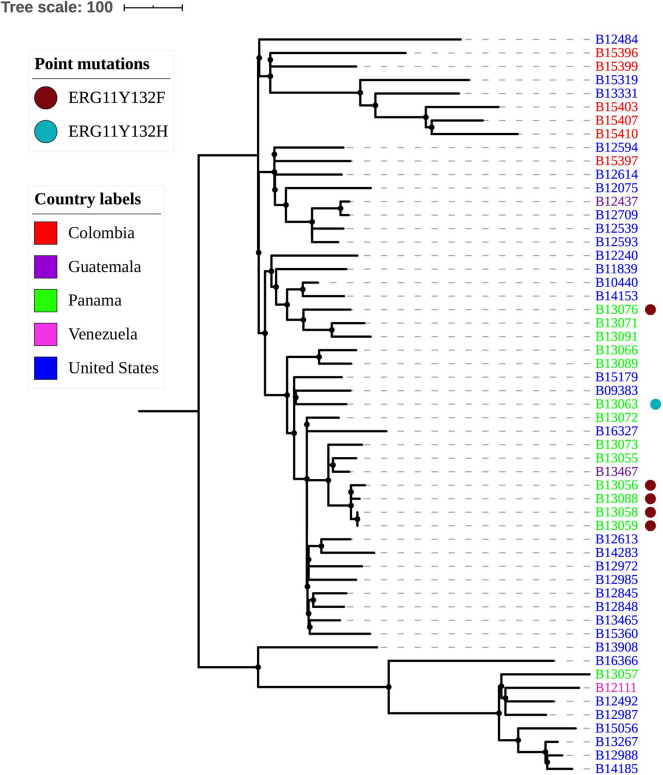
Genetic relationships among *C. duobushaemulonii* isolates. The maximum parsimony tree was constructed using 6614 SNPs called against B09383 *C. duobushaemulonii* reference genome (GenBank accession PKFP00000000). Scale bar shows pairwise SNPs.

The average pairwise distance between *C. duobushaemulonii* isolates was 458 SNPs (range 0–1,243). The isolates can be separated into two genetically distinct clades separated by more than 600 SNPs. One of these clades included isolates from Panama, Colombia, Guatemala, Venezuela, and the United States, and the other included primarily isolates from the United States, one isolate from Venezuela, and one from Panama (Figure 3 and https://microreact.org/project/Candida_duobushaemulonii).

Nine of the 14 isolates from Panama were from a previously reported outbreak in a large hospital in Panama City (Araúz et al., 2018); five of those were genetically distinct and intermixed with isolates from other regions, and four others, each isolated from a different patient, formed a tight cluster on the phylogenetic tree (Figure 3). B13058 from a toenail and B13059 from blood were isolated in November 2016 and were identical (0 SNPs), and the other two, B13056 from the catheter isolated in November 2016 and B13088 from blood isolated in April 2017, were separated by <42 SNPs. Clusters of *C. duobushaemulonii* and *C. haemulonii* overlapped in time. All *C. duobushaemulonii* isolates were mating type alpha. The genome-wide nucleotide diversity (π) was 4.86e-05, which was almost twice that observed in *C. haemulonii*; however, the average Tajima's D estimate was −1.17, which was similar to that of *C. haemulonii* and consistent with recent population expansion.

### Genetic Relationships Among Isolates of *C. pseudohaemulonii* and *C. vulturna*

**Figure 4:**
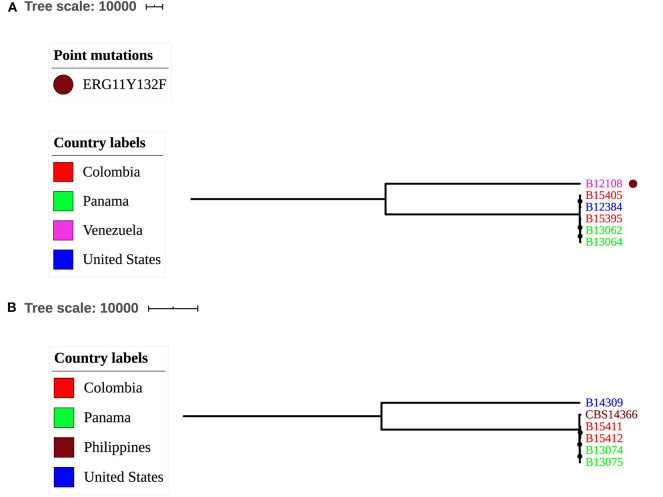
Genetic relationships among *C. pseudohaemulonii* **(A)** and *C. vulturna* **(B)** isolates. Maximum parsimony trees were constructed using a total of 238,026 SNPs called against B12108 *C. pseudohaemulonii* reference genome (GenBank accession PKFO000000000) and 81,034 SNPs called against CBS14366 *C. vulturna* reference assembly Bio project PRJNA560499. Scale bar shows pairwise SNPs.

Five of the six tested isolates of *C. pseudohaemulonii* clustered together (441-845 SNPs), and the sixth isolate, B12108 from blood of a Venezuelan patient, was >200,000 SNPs different from the other group (Figure 4A and https://microreact.org/project/Candida_pseudohaemulonii). Similarly, four isolates of *C. vulturna* from Colombia, Panama, and Venezuela were different from each other and the type isolate, CBS14366, isolated from a flower in the Philippines (Sipiczki and Tap, 2016) by 104–209 SNPs. However, the fifth isolate from a U.S. patient was more than 81,000 SNPs different from the other four, suggesting multiple clades of *C. vulturna* (Figure 4B and https://microreact.org/project/Candida_vulturna) although more isolates are needed to test this hypothesis. All *C. pseudohaemulonii* isolates were mating type a. In *C. vulturna*, four isolates where mating type a, and the fifth isolate from the United States (B14309) was mating type alpha.

### Antifungal Susceptibility Testing and Mutations Linked to Resistance

We observed variable levels of susceptibility to amphotericin B among 38 tested *C. haemulonii* isolates: 29 (76%) had elevated MICs from 2 μg/mL to >32 μg/mL, and the rest had MICs below 2 μg/mL (Table 3). Seven (18%) isolates had elevated MIC of fluconazole ranging from 32 to 256 μg/mL, and all had the Y132F mutation in *ERG11*. However, one isolate, B13067, with this mutation had MIC of fluconazole 8 μg/mL (https://microreact.org/project/Candida_haemulonii).

**Table 3:** Antifungal susceptibility profiles and *ERG11* point mutations.

**Strain or Isolate**	**MIC (μg/mL)**									***ERG11*** **point mutations**			
	**Broth microdilution**								**E test**				
	**VOR**	**AND**	**CAS**	**FZ**	**IZ**	**ISA**	**PZ**	**MF**	**AMB**				
***C. haemulonii***													
B10441	0.03	0.06	0.03	8	0.06	0.016	<0.008	0.016	1.5				
B11786	0.125	0.06	0.06	64	0.5	0.125	<0.008	0.03	1	Y132F			
B11792	0.03	0.06	0.06	4	0.125	0.03	<0.008	0.03	16				
B11798	0.03	0.06	0.06	128	0.5	0.125	<0.008	0.06	3	Y132F			
B11803	0.06	0.06	0.03	64	0.06	0.03	<0.008	0.03	0.75	Y132F			
B11898	0.016	0.125	0.125	4	0.25	0.03	0.03	0.125	12				
B11899	<0.008	0.25	0.125	4	0.5	0.125	0.06	0.06	1.5				
B11900	0.03	0.06	0.03	8	0.06	0.016	<0.008	0.016	0.75				
B12068	0.06	0.125	0.125	16	0.5	0.5	0.25	0.06	4				
B12109	0.06	0.06	0.03	8	0.06	0.03	<0.008	0.03	2				
B12112	<0.008	0.03	1	256	0.5	0.25	0.016	0.03	0.75	Y132F			
B12126	NG	0.03	0.06	8	0.5	0.03	0.03	0.03	0.125				
B12127	<0.008	0.125	16	256	2	1	0.5	0.016	24	Y132F			
B12128	NG	0.125	0.06	2	0.25	0.016	0.016	0.125	6				
B12129	0.03	0.03	0.06	4	0.06	0.016	<0.008	0.03	1.5				
B12185	<0.008	0.125	0.06	2	0.06	0.008	0.016	0.06	2				
B12201	<0.008	0.03	0.03	4	0.5	0.03	0.03	0.06	1				
B12343	0.03	0.06	0.03	4	0.5	0.06	0.125	0.06	3				
B12615	0.03	0.06	0.125	4	0.25	0.06	0.03	0.06	0.38				
B12643	0.06	0.06	0.06	8	0.25	0.016	0.016	0.06	1.5				
B12989	0.016	0.03	0.03	2	0.016	<0.04	NG	0.016	12				
B13065	0.03	0.03	0.03	4	0.5	0.03	0.03	0.06	3				
B13067	0.06	0.06	0.03	8	0.06	0.03	0.016	0.03	32	Y132F			
B13068	0.03	0.06	0.03	32	0.03	0.016	0.016	<0.008	4	Y132F			
B13081	0.03	0.03	0.03	32	0.03	0.008	<0.008	0.016	1	Y132F			
B13273	<0.008	0.06	0.03	2	9.25	0.008	0.016	0.03	12				
B13444	0.03	0.06	0.03	4	0.25	0.03	0.03	0.03	16				
B13704	0.03	0.03	0.03	4	0.5	0.03	0.06	0.03	12				
B13909	0.03	0.06	0.06	4	0.5	0.03	0.03	0.06	>32				
B15318	0.03	0.125	0.06	4	0.5	0.03	0.06	0.125	>32				
B15393	0.03	0.03	0.03	4	0.5	0.06	0.125	0.06	3				
B15394	0.03	0.06	0.06	4	0.25	0.03	0.016	0.06	16				
B15400	0.125	0.125	0.03	16	0.5	0.25	0.25	0.06	2				
B15401	0.03	0.25	0.125	4	0.125	0.016	0.016	0.06	3				
B15406	0.06	0.03	0.06	8	0.25	0.03	0.03	0.06	4			·	
B15408	0.03	0.06	0.06	2	0.125	0.016	<0.008	0.03	0.75				
B15409	0.03	0.125	0.06	4	0.125	0.03	<0.008	0.06	3				
B16299	0.03	0.06	0.03	8	0.125	0.06	0.016	0.06	1.5				
***C. duobushaemulonii***													
B09383	0.06	0.016	0.016	8	0.125	0.03	0.06	0.06	>32				
B10440	0.06	0.06	0.03	8	0.125	0.03	<0.008	0.06	16		·		
B11839	0.06	0.125	0.125	8	0.25	0.06	0.016	0.06	32				
B12075	0.03	0.06	0.125	8	0.25	0.016	0.03	0.06	12				
B12111	0.06	0.06	0.03	8	0.06	0.03	<0.008	0.03	32				
B12240	0.125	0.03	0.03	16	1	0.125	0.25	0.016	0.064				
B12437	0.03	1	0.25	2	0.25	0.016	0.03	0.25	0.25				
B12484	0.5	0.25	0.06	16	1	0.25	0.25	0.06	>32				
B12492	0.125	0.25	0.125	4	0.25	0.03	0.125	0.06	>32				
B12539	0.06	0.06	0.06	4	0.03	0.03	0.125	0.125	>32				
B12593	0.06	0.125	0.06	16	0.06	0.03	0.125	0.125	>32				
B12594	0.06	0.06	0.03	4	0.25	0.03	0.06	0.06	32				
B12613	1	0.5	0.25	16	0.5	0.5	0.5	0.125	>32				
B12614	0.06	0.125	0.125	16	0.25	0.06	0.016	0.06	24				
B12709	0.06	0.5	0.125	16	0.5	0.25	0.25	0.125	>32				
B12845	0.125	0.25	0.06	8	0.5	0.125	0.125	0.125	32				
B12848	0.06	0.125	0.06	8	0.5	0.03	0.06	0.06	>32				
B12972	0.06	0.06	0.125	16	0.5	0.125	0.125	0.06	32				
B12985	0.03	0.25	0.25	4	0.06	0.03	0.016	0.03	32				
B12987	0.06	0.25	0.125	16	0.5	0.06	0.06	0.125	24				
B12988	0.06	0.25	0.06	16	0.5	0.06	0.125	0.125	32				
B13055	0.03	0.06	0.06	8	0.125	0.03	0.016	0.03	32				
B13056	2	0.25	0.06	>256	0.5	0.125	0.03	0.03	24	Y132F			G307A
B13057	0.03	0.125	0.03	8	0.125	0.03	<0.008	0.03	16				
B13058	1	0.125	0.125	256	0.25	0.06	0.03	0.03	32	Y132F			G307A
B13059	2	0.125	0.125	256	0.25	0.06	0.03	0.03	32	Y132F			G307A
B13063	8	0.125	0.06	256	2	2	0.25	0.06	24		Y132H	G443D	
B13066	0.03	0.03	0.03	8	0.06	0.016	0.016	0.016	12				
B13071	0.03	0.125	0.06	8	0.06	0.016	<0.008	0.06	>32				
B13072	0.25	1	0.25	16	0.25	0.06	0.016	0.06	>32				
B13073	0.06	0.25	0.125	16	0.5	0.06	0.06	0.06	32				
B13076	1	0.06	0.03	256	0.25	0.06	0.016	0.03	32	Y132F			
B13088	2	1	0.25	256	0.5	0.125	0.03	0.06	16	Y132F			G307A
B13089	0.06	0.03	0.03	8	0.5	0.03	0.06	0.06	24				
B13091	0.03	0.06	0.03	2	0.125	0.016	0.016	0.06	16				
B13267	0.06	0.25	0.06	8	0.25	0.03	0.03	0.06	12				
B13331	0.125	0.125	0.06	16	0.5	0.06	0.03	0.06	32				
B13465	0.06	0.03	0.06	16	0.125	0.016	0.016	0.03	24				
B13467	0.06	0.125	0.06	4	0.25	0.03	0.03	0.06	0.25				
B13908	0.06	0.25	0.06	8	0.5	0.03	0.03	0.06	32				
B14153	0.03	0.125	0.03	8	0.25	0.03	0.03	0.03	>32				
B14185	0.03	0.06	0.03	8	0.125	0.016	0.016	0.03	0.38				
B14283	0.06	0.125	0.125	16	0.125	0.03	<0.008	0.06	12				
B15056	0.06	0.25	0.125	16	0.5	0.06	0.25	0.125	>32				
B15179	0.125	0.06	0.06	8	0.5	0.06	0.06	0.06	32				
B15319	0.03	0.125	0.125	8	0.25	0.03	0.03	0.125	>32				·
B15360	0.5	2	0.5	64	2	1	0.5	0.25	32				
B15396	0.03	0.06	0.03	8	0.25	0.03	0.03	0.06	16				
B15397	0.06	0.125	0.016	8	0.5	0.03	0.06	0.06	>32				
B15399	0.06	0.5	0.125	8	0.5	0.03	0.125	0.06	>32				
B15403	0.06	0.06	0.03	8	0.25	0.06	0.03	0.125	32				
B15407	0.06	0.03	0.06	8	0.25	0.03	0.03	0.06	32				
B15410	0.016	0.25	0.25	16	0.25	0.06	0.016	0.06	32				
B16327	0.06	0.25	0.125	8	0.5	0.06	0.125	0.06	>32				
B16366	0.03	0.06	0.03	8	0.25	0.03	0.016	0.03	12				
***C. pseudohaemulonii***													
B12108	1	0.06	0.016	128	0.125	0.016	<0.008	0.03	12	Y132F			
B12384	0.125	0.125	0.03	32	0.25	0.125	0.03	0.06	>32				
B13062	0.03	0.125	0.03	2	0.016	0.008	<0.008	0.03	0.125				
B13064	0.03	0.06	0.06	8	0.06	0.016	NG	0.03	0.38				
B15395	0.06	0.06	0.06	8	0.06	0.06	<0.008	0.06	0.125				
B15405	0.03	0.25	0.03	8	0.06	0.03	<0.008	0.06	0.125				
***C. vulturna***													
B13074	0.03	0.5	16	8	0.06	0.125	0.016	0.06	24				
B13075	0.125	0.03	0.03	16	0.5	0.03	0.125	0.03	16				
B14309	0.06	0.06	0.03	8	0.5	0.03	0.06	0.06	>32				
B15411	0.06	0.06	0.06	16	0.5	0.5	0.125	0.03	8				
B15412	0.06	0.125	0.06	8	0.25	0.125	0.03	0.03	12				

*MIC, minimum inhibitory concentration; VOR, Voriconazole; AND, Anidulafungin; CAS, Caspofungin; FZ, Fluconazole; IZ, Itraconazole; ISA, Isavuconazole; PZ, Posaconazole; MF, Micafungin; AMB, Amphotericin B; NG, No growth*.

Three *C. haemulonii* isolates, B11786, B11792, and B11803, had aneuploidy of chromosome 4 (scaffold 4) that harbors *ERG11*: B11786 and B11792 have complete duplication of chromosome 4, and B11803 has a 300-kb duplication or a region encompassing *ERG11* (Supplementary Figure 1). In addition to this duplication, both B11786 and B11803 had Y132F substitution, and their MIC of fluconazole was 64 μg/mL. However, the MIC of fluconazole of B11792 that had wild-type *ERG11* copy was 4 μg/mL, suggesting that the duplication alone did not increase resistance (Table 3).

Of the 55 *C. duobushaemulonii* isolates, 51 (93%) had highly elevated MICs of amphotericin B ranging from 12 μg/mL to more than 32 μg/mL (Table 3). Four isolates (7%), two from Guatemala (B13467 and B12437). and two from the United States (B12240 and B14185), had lower MICs (0.064–0.38) of amphotericin B although all four were genetically unrelated to each other (Figure 1). Conversely, 48 of 55 (87%) *C. duobushaemulonii* had lower MICs (≤16 μg/mL) of fluconazole, and seven (13%) had elevated MIC (≥64 μg/mL). Of those seven, five with MIC of 256 μg/mL had the Y132F mutation in the *ERG11* gene, and they also had elevated MIC of voriconazole (1–2 μg/mL) (Table 3). Furthermore, four of those isolates, which formed a tight cluster associated with an outbreak in Panama (Figure 3), also had G307A substitution, whereas B13063 had Y132H and G443D substitutions in *ERG11* and had MIC of fluconazole of 64 μg/mL (https://microreact.org/project/Candida_duobushaemulonii). All four of these mutations have been linked to resistance to fluconazole in *C. albicans* (Berkow and Lockhart, 2017).

Of the six tested *C. pseudohaemulonii* isolates, two, B12108 and B12384, had elevated MICs of amphotericin B, 12 μg/mL and >32 μg/mL, and the same two isolates had elevated MICs of fluconazole of 128 and 32 μg/mL, respectively (Table 3). Of those, B12108 had the Y132F substitution (Muñoz et al., 2018).

All five tested *C. vulturna* isolates had elevated MICs of amphotericin B with MICs ranging from 8 μg/mL to more than 16 μg/mL (Table 3). All five isolates were susceptible to fluconazole with MIC of 8 to 16 μg/mL, and no known substitutions in the *ERG11* gene were detected in these isolates (Table 3). All tested isolates of the four species had low MICs of echinocandins.

In addition, we examined other genes that have been implicated in azole and amphotericin B resistance in *Candida* s*pp*. (Arendrup and Patterson, 2017); the non-synonymous substitutions identified in these genes are listed in Supplementary Table 2. The annotated VCF files for all isolates from this study that can be used to browse for substitutions in other genes of interest are available as https://figshare.com/projects/Genome_sequencing_of_Candida_haemulonii_species_complex/80150.

## Discussion

We present results of the population genetics and phylogenetic analyses of isolates from the *Candida haemulonii* complex that are closely related to the globally emerging pathogen, *C. auris*. Phylogenetic analysis based on WGS confirmed previously described relationships among these species (Muñoz et al., 2018; Navarro-Muñoz et al., 2019) and demonstrated that the newly described *C. vulturna* forms a distinct, well-supported clade related to *C. duobushaemulonii* and *C. pseudohaemulonii*. Here, we describe five additional clinical isolates of this new species and demonstrate that it can cause both invasive as well as non-invasive infections as isolates of this species were isolated from blood, wounds, and other body fluids. This new species was found in Panama, Colombia, and in the United States.

Isolates of *C. haemulonii* and *C. duobushaemulonii* were recovered from blood as well as from wounds and other non-invasive sites. In the United States, the vast majority of *C. haemulonii* and *C. duobushaemulonii* were from non-invasive sites, such as lower extremities or wounds, and most isolates from Latin America were from blood. Many of the isolates from the United States were described as from foot or toe wounds, so it is possible that these species may be associated with diabetic foot infections although more epidemiological data is needed to show a definitive link. The reasons for this observed difference between the clinical presentations of the infections in the United States and Latin America are not entirely clear. One possible explanation may be that, compared to the United States, fewer *Candida* from non-invasive sites are collected and identified in Latin America; therefore, isolates from the non-invasive sites may be less likely to be reported. However, it is also possible that different infection control or clinical practices in Latin America led to more *C. haemulonii* and *C. duobushaemulonii* invasive infections. Notably, almost all azole-resistant isolates of *C. haemulonii* and *C. duobushaemulonii* were found in Latin America. Regardless of their geographic origin, all isolates of *C. pseudohaemulonii* were from blood. Whether this is truly a difference in the epidemiology among the species remains to be determined as only a few isolates of this species were available.

Population genetic analysis demonstrated that most isolates of *C. haemulonii, C. duobushaemulonii, C. pseudohaemulonii*, and *C. vulturna* were genetically distinct and do not yet show evidence of the extensive clonality that can be a sign of global emergence. However, we identified several nearly identical isolates of *C. duobushaemulonii* and *C. haemulonii* in different patients in one hospital in Panama, which reported outbreaks of *C. duobushaemulonii* and *C. auris* (Araúz et al., 2018). This finding indicates that both species are capable of transmission and causing outbreaks in healthcare settings. The reasons for the simultaneous outbreaks of *C. auris, C. haemulonii*, and *C. duobushaemulonii* in this hospital are unknown. Likely factors include a large patient population and poor infection control practices. It is also possible that some unique clinical practices in this facility, such as extensive use of azoles, might have contributed to multiple outbreaks. Notably, all *C. haemulonii* and *C. duobushaemulonii* from the outbreaks in this hospital were carrying *ERG11* mutations, and most had elevated MICs to fluconazole.

The overall genomic diversity (π) in the *C. haemulonii* population was relatively low and comparable with that observed in different clades of *C. auris*; no phylogeographic population substructure was observed. Although isolates of *C. haemulonii* var. *vulnera* were identified by others based on sequencing the ITS region of rDNA (Cendejas-Bueno et al., 2012), we were unable to confirm this observation with genomic data. Indeed, three isolates identified by MALDI-TOF as *C. haemulonii* var. *vulnera* were indistinguishable from other *C. haemulonii* strains, suggesting that databases need to be updated to correctly identify all *C. haemulonii*. Whereas, both mating types have been reported in different clades of *C. auris*, all *C. haemulonii* isolates were mating type alpha. The 58-year-old B10441 (CBS 5149) isolate recovered from fish in 1962 was not notably different from other contemporaneous strains, suggesting low genetic diversity in this species.

Compared with *C. haemulonii*, more diversity was observed among *C. duobushaemulonii* isolates: Two subclades separated by more than 600 SNPs were observed. The genomic diversity (π) in *C. duobushaemulonii* was almost twice that of *C. haemulonii*, which was probably reflective of the more complex population structure. However, the genome-wide Tajima's D estimates for both species were negative and consistent with clonally expanding populations. Comparable estimates of Tajima's D were obtained for three rapidly expanding clades of *C. auris* (Chow et al., 2020). Furthermore, similarly to *C. haemulonii*, all *C. duobushaemulonii* isolates in our study were also mating type alpha.

Antifungal susceptibility testing indicated that most isolates from *C. haemulonii* species complex had highly elevated MICs of amphotericin B. Specifically, 100% of *C. vulturna*, 93% of *C. duobushaemulonii*, 76% of *C. haemulonii*, and 33% of *C. pseudohaemulonii* showed elevated MICs of at least 2 μg/mL. Elevated MIC values of fluconazole were also common but not nearly as widespread as in *C. auris*. Approximately 13% of *C. duobushaemulonii*, 18% of *C. haemulonii*, and 33% of *C. pseudohaemulonii* had MICs of fluconazole of 32 μg/mL or higher, and all isolates of *C. vulturna* were susceptible to fluconazole. All isolates of all four species had low MIC values of echinocandins.

The majority of isolates with elevated MICs of fluconazole in *C. haemulonii, C. duobushaemulonii*, and *C. pseudohaemulonii* contained the Y132F substitution in *ERG11*, which is associated with azole resistance in *C. albicans* and *C. auris* Clades I and IV (Berkow and Lockhart, 2017; Muñoz et al., 2018). Furthermore, additional substitutions linked to azole resistance in *C. albicans* were identified in *C. duobushaemulonii* isolates from Panama that also had Y132F/H substitutions; however, it was unclear whether these additional substitutions further affected MIC levels of azoles. No previously described drug-related mutations in the *ERG11* gene (Berkow and Lockhart, 2017) were detected in *C. vulturna* isolates with elevated MICs, suggesting a different mechanism of resistance. All *C. haemulonii* and *C. duobushaemulonii* with elevated MICs of fluconazole were found in Colombia, Venezuela, and Panama. Only a single isolate of *C. pseudohaemulonii* with a moderately high MIC of fluconazole of 32 μg/mL was isolated in the U.S. state of Georgia, and no other fluconazole-resistant isolates were found in the United States despite testing a comparable number of isolates from the United States and Latin America. In addition, all isolates with the corresponding *ERG11* mutations and chromosomal duplications that can be linked to azole resistance were found only in Latin America. Additional non-synonymous substitutions were detected in other genes implicated in antifungal resistance in *Candida* (Arendrup and Patterson, 2017); however, more research is needed to evaluate the role of these substitutions in resistance to antifungal drugs. The list of the non-synonymous substitutions and annotated VCF files is provided in our study for other investigators who might be interested in addressing these questions (https://figshare.com/projects/Genome_sequencing_of_Candida_haemulonii_species_complex/80150 and Supplementary Table 2).

Our results indicate that, although we are not yet observing the widespread emergence of fungi for the *C. haemulonii* species complex as human pathogens, at least two of these species can be transmitted within a healthcare facility and may cause healthcare-associated outbreaks. Outbreak isolates of both species also had elevated MICs of azoles and had corresponding *ERG11* mutations and chromosomal duplications linked to resistance. The prevalence of these mutations and duplications among *C. haemulonii* and *C. duobushaemulonii* isolates from Latin America suggests that these species may be exposed to the same evolutionary pressure from azole drugs that may have contributed to the emergence of *C. auris*. Continued surveillance to monitor azole resistance and epidemiology of these species is warranted.

## Data Availability Statement

All whole genome sequence raw reads for this study can be found in the NCBI under the following Bio Project PRJNA606185.

## Ethics Statement

The studies involving human participants were reviewed and approved by CDC's IRB-Committee 2. Written informed consent for participation was not required for this study in accordance with the national legislation and the institutional requirements.

## Author Contributions

LG and MS generated data. LG, JM, DW, and KF performed the analysis. EB, BJ, CC, and AL supervised the study. DC, KF, RR-C, PE, MD, RB-A, and AE-B provided the materials. LG, JM, and AL wrote the manuscript. LG, SL, and AL conceived the project.

## Conflict of Interest

DW was employed by the company IHRC, Inc. The remaining authors declare that the research was conducted in the absence of any commercial or financial relationships that could be construed as a potential conflict of interest.

## References

[B1] Araúz A. B. Caceres D. H. Santiago E. Armstrong P. Arosemena S. Ramos C. (2018). Isolation of *Candida auris* from 9 patients in Central America: importance of accurate diagnosis and susceptibility testing. Mycoses 61, 44–47. 10.1111/myc.12709 28945325

[B2] Arendrup M. C. Patterson T. F. (2017). Multidrug-resistant *Candida*: epidemiology, molecular mechanisms, and treatment. J. Infect. Dis. 216(Suppl. 3), S445–S451. 10.1093/infdis/jix131 28911043

[B3] Ben-Ami R. Berman J. Novikov A. Bash E. Shachor-Meyouhas Y. Zakin S.. (2017). Multidrug-resistant *Candida haemulonii* and *C. auris, Tel Aviv, Israel*. Emerg. Infect. Dis. 23:195. 10.3201/eid2302.161486 28098529 PMC5324804

[B4] Berkow E. L. Lockhart S. R. (2017). Fluconazole resistance in *Candida* species: a current perspective. Infect. Drug Resist. 10:237. 10.2147/IDR.S118892 28814889 PMC5546770

[B5] Cendejas-Bueno E. Kolecka A. Alastruey-Izquierdo A. Theelen B. Groenewald M. Kostrzewa M. (2012). Reclassification of the *Candida haemulonii* complex as *Candida haemulonii* (*C. haemulonii group I), C. duobushaemulonii sp. nov. (C. haemulonii group II), and C. haemulonii var. vulnera var. nov.:* three *multiresistant human pathogenic yeasts*. J. Clin. Microbiol. 50, 3641–3651. 10.1128/JCM.02248-12 22952266 PMC3486233

[B6] Chow N. A. Gade L. Batra D. Rowe L. A. Juieng P. Ben-Ami R.. (2018a). Genome sequence of a multidrug-resistant *Candida haemulonii* isolate from a patient with chronic leg ulcers in Israel. Genome Announc. 6, e00176-18. 10.1128/genomeA.00176-18 29650567 PMC5897814

[B7] Chow N. A. Gade L. Batra D. Rowe L. A. Juieng P. Loparev V. N.. (2018b). Genome sequence of the amphotericin B-resistant *Candida duobushaemulonii* strain B09383. Genome Announc. 6:e00204-18. 10.1128/genomeA.00204-18 29599161 PMC5876483

[B8] Chow N. A. Muñoz J. F. Gade L. Berkow E. L. Welsh R. M. Forsberg K.. (2020). Tracing the evolutionary history and global expansion of *Candida auris* using population genomic analyses. MBio 11, e03364-19. 10.1128/mBio.03364-19 32345637 PMC7188998

[B9] Chowdhary A. Sharma C. Duggal S. Agarwal K. Prakash A. Singh P. K.. (2013). New clonal strain of *Candida auris, Delhi, India*. Emerg. Infect. Dis. 19, 1670–1673. 10.3201/eid1910.130393 24048006 PMC3810747

[B10] CLSI (2008). Reference Method for Broth Dilution Antifungal Susceptibility Testing of Yeasts: Approved Standard, 3rd Edn, M27-A3 Wayne, PA: Clinical and Laboratory Standards Institute.

[B11] Gargeya I. B. Pruitt W. R. Meyer S. A. Ahearn D. G. (1991). *Candida haemulonii* from clinical specimens in the USA. J. Med. Vet. Mycol. 29, 335–338. 10.1080/02681219180000511 1955954

[B12] Hou X. Xiao M. Chen S. C. Wang H. Cheng J. W. Chen X. X.. (2016). Identification and antifungal susceptibility profiles of *Candida haemulonii* species complex clinical isolates from a multicenter study in China. J. Clin. Microbiol. 54, 2676–2680. 10.1128/JCM.01492-16 27535688 PMC5078542

[B13] Isla G. Taverna C. G. Szusz W. Vivot W. García-Effron G. Davel G. (2017). *Candida haemulonii sensu lato*: update of the Determination of Susceptibility Profile in Argentina and Literature Review. Curr Fung Infect Rep. 11, 203–208. 10.1007/s12281-017-0300-y

[B14] Jackson B. R. Chow N. Forsberg K. Litvintseva A. P. Lockhart S. R. Welsh R.. (2019). On the origins of a species: what might explain the rise of *Candida auris*? J Fung. 5:58. 10.3390/jof5030058 31284576 PMC6787658

[B15] Khan Z. U. Al-Sweih N. A. Ahmad S. Al-Kazemi N. Khan S. Joseph L.. (2007). Outbreak of fungemia among neonates caused by *Candida haemulonii* resistant to amphotericin B, itraconazole, and fluconazole. J. Clin. Microbiol. 45, 2025–2027. 10.1128/JCM.00222-07 17428940 PMC1933024

[B16] Kumar S. Stecher G. Li M. Knyaz C. Tamura K. (2018). MEGA X: molecular evolutionary genetics analysis across computing platforms. Mol. Biol. Evol. 5, 1547–1549. 10.1093/molbev/msy096 29722887 PMC5967553

[B17] Kurtz S. Phillippy A. Delcher A. L. Smoot M. Shumway M. Antonescu C.. (2004). Versatile and open software for comparing large genomes. Genome Biol. 5:R12. 10.1186/gb-2004-5-2-r12 14759262 PMC395750

[B18] Letunic I. Bork P. (2019). Interactive Tree Of Life (iTOL) v4: recent updates and new developments. Nucleic Acids Res. 47, W256–W259. 10.1093/nar/gkz239 30931475 PMC6602468

[B19] Li H. Durbin R. (2009). Fast and accurate short read alignment with Burrows–Wheeler transform. Bioinformatics 25, 1754–1760. 10.1093/bioinformatics/btp324 19451168 PMC2705234

[B20] Muñoz J. F. Gade L. Chow N. A. Loparev V. N. Juieng P. Berkow E. L.. (2018). Genomic insights into multidrug resistance, mating and virulence in *Candida auris* and related emerging species. Nat. Commun. 9, 1–3. 10.1038/s41467-018-07779-6 30559369 PMC6297351

[B21] Navarro-Muñoz J. C. de Jong A. W. van den Ende B. G. Haas P. J. Then E. R. Tap R. M.. (2019). The high-quality complete genome sequence of the opportunistic fungal pathogen *Candida vulturna* CBS 14366 T. Mycopathologia 84, 731–734. 10.1007/s11046-019-00404-0 31734799

[B22] Ramos L. S. Figueiredo-Carvalho M. H. Barbedo L. S. Ziccardi M. Chaves A. L. Zancopé-Oliveira R. M.. (2015). *Candida haemulonii* complex: species identification and antifungal susceptibility profiles of clinical isolates from Brazil. J. Antimicrob. Chemother. 70, 111–115. 10.1093/jac/dku321 25134720

[B23] Satoh K. Makimura K. Hasumi Y. Nishiyama Y. Uchida K. Yamaguchi H. (2009). Candida auris sp. nov., a novel ascomycetous yeast isolated from the external ear canal of an inpatient in a Japanese hospital. Microbiol Immunol. 53, 41–44. 10.1111/j.1348-0421.2008.00083.x 19161556

[B24] Sipiczki M. Tap R. M. (2016). *Candida vulturna* pro tempore sp. nov., a dimorphic yeast species related to the Candida haemulonis species complex isolated from flowers and clinical sample. Int. J. Syst. Evol. Microb. 66, 4009–4015. 10.1099/ijsem.0.001302 27411802

[B25] Van Uden N. Kolipinski M. C. (1962). Torulopsis haemulonii nov. spec. a yeast from the Atlantic Ocean. Antonie Van Leeuwenhoek 28, 78–80. 10.1007/BF02538724 14039875

