# Supplementary material for: Understanding the Emergence of Multidrug-Resistant Candida: Using Whole-Genome Sequencing to Describe the Population Structure of Candida haemulonii Species Complex
Source: Front Genet. 2020 Jun 10;11:554. doi: 10.3389/fgene.2020.00554 (PMC7298116; doi:10.3389/fgene.2020.00554)
Supplement: Supplementary file 1 [file Image_1.pdf]

Supplementary Material

Supplementary Figure 1. Copy number variation (CNV) analysis in 38 isolates of *Candida haemulonii* using B11899 as reference. Each scaffold from 1 to 11 is color-coded in the *x*-axis. Average normalized depth (5-kb windows) on the *y*-axis is relative to ploidy levels.

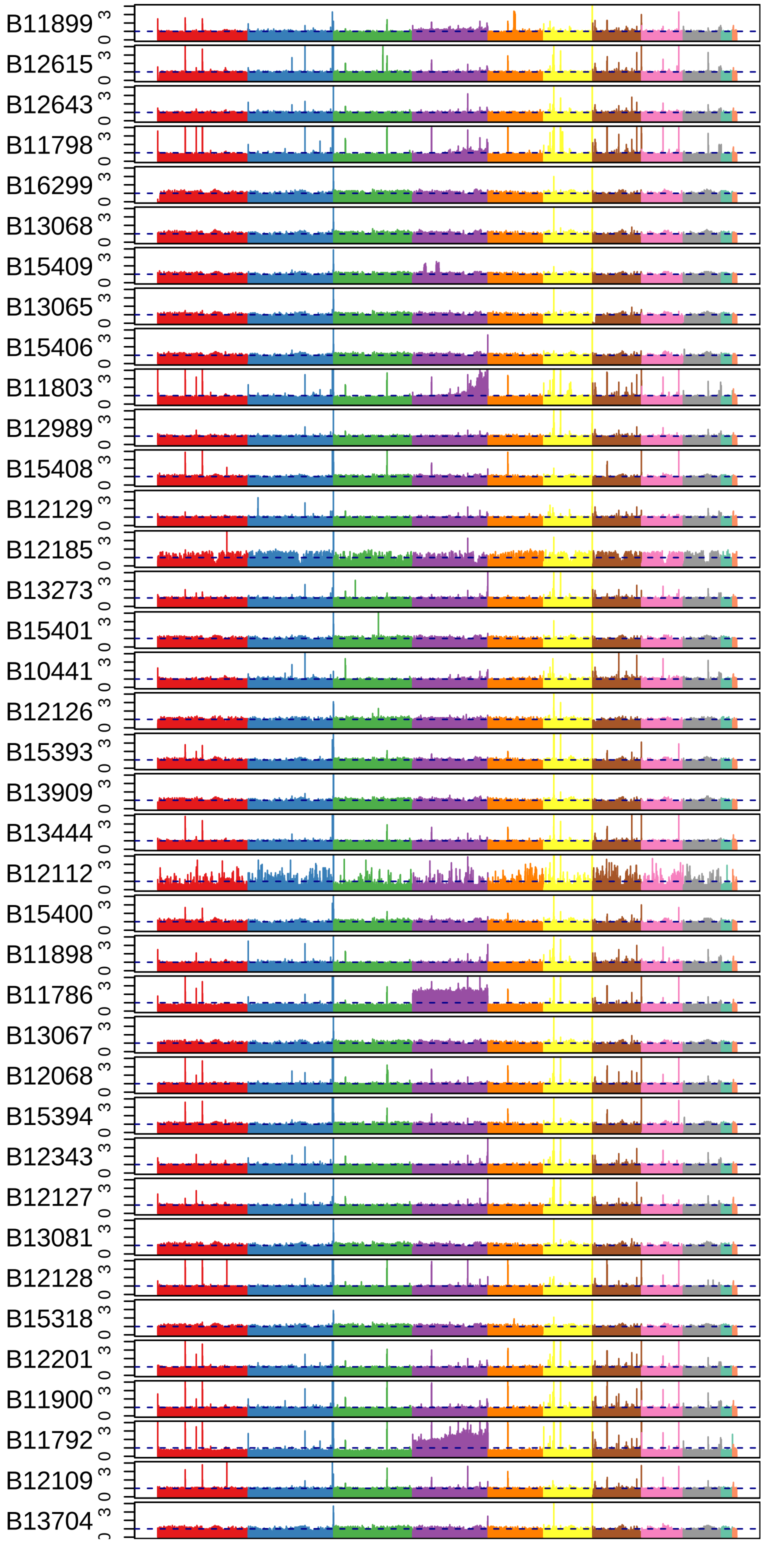
B11899
B12615
B12643
B11798
B16299
B13068
B15409
B13065
B15406
B11803
B12989
B15408
B12129
B12185
B13273
B15401
B10441
B12126
B15393
B13909
B13444
B12112
B15400
B11898
B11786
B13067
B12068
B15394
B12343
B12127
B13081
B12128
B15318
B12201
B11900
B11792
B12109
B13704
